# Combining antibody conjugates with cytotoxic and immune‐stimulating payloads maximizes anti‐cancer activity

**DOI:** 10.1002/1878-0261.70198

**Published:** 2026-01-06

**Authors:** Tiexin Wang, Dong Jun Koo, Peter M. Tessier, Greg M. Thurber

**Affiliations:** ^1^ Department of Chemical Engineering University of Michigan Ann Arbor USA; ^2^ Biointerfaces Institute, University of Michigan Ann Arbor USA; ^3^ Department of Biomedical Engineering University of Michigan Ann Arbor USA; ^4^ Department of Pharmaceutical Sciences University of Michigan Ann Arbor USA; ^5^ Program in Chemical Biology University of Michigan Ann Arbor USA; ^6^ Rogel Cancer Center University of Michigan Ann Arbor USA

**Keywords:** ADC, antibody–drug conjugate, CEA, immune‐stimulating antibody conjugate, ISAC, STING agonist

## Abstract

Antibody–drug conjugates (ADCs) are rapidly expanding in the clinical treatment of cancers, and combinations with checkpoint inhibitors further enhance antitumor activity in patients sensitive to such immunotherapy. However, a method to improve treatment durability, including cases where immunologically cold tumors limit checkpoint inhibitor activity, is needed. Here, we demonstrate that mixtures of ADCs and immune‐stimulating antibody conjugates (ISACs) enhance efficacy compared to either agent alone. Our approach utilizes two non‐competitive antibodies to increase the internalization of a tumor‐associated antigen (carcinoembryonic antigen, CEA), facilitating the entry of the toxic payload (SN‐38, a topoisomerase I inhibitor) into cancer cells. With improved FcγR engagement, the designed ISAC better delivered the immunostimulatory agent (STING agonist) into immune cells. After treatment, the average tumor volume in the combination group was ~40% of the ADC group, and ~30% of the PBS group at day 14. The side effects of combination therapy were tolerable, with an average weight loss of 7% or less after injections. We expect this approach can be readily extended to other ADCs to enhance their efficacy, including for the treatment of immunologically cold tumors.

## Graphical abstract

Methods to improve antibody–drug conjugate (ADC) treatment durability in cancer therapy are needed. We utilized ADCs and immune‐stimulating antibody conjugates (ISACs), which are made from two non‐competitive antibodies, to enhance the entry of toxic payloads into cancer cells and deliver immunostimulatory agents into immune cells. The combination exhibited enhanced efficacy compared to either agent alone with tolerable side effects in mice.

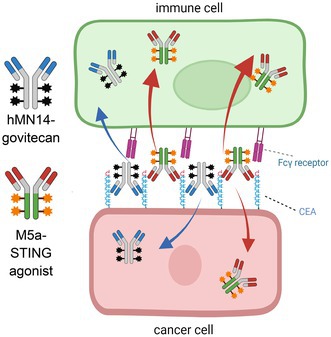

**Abbreviations**
ADC: antibody–drug conjugates
ADCC: antibody‐dependent cellular cytotoxicity
ADCP: antibody‐dependent cellular phagocytosis
CEA: carcinoembryonic antigen
DAR: drug‐to‐antibody ratio
HER2: human epidermal growth factor receptor 2
HI FBS: fetal bovine serum, heat inactivated
ISAC: immune‐stimulating antibody conjugate
PBS: phosphate‐buffered saline
STING: stimulators of interferon genes
TLR: toll‐like receptor
Trop2: trophoblast cell surface antigen 2

## 1 Introduction

Antibody–drug conjugates (ADCs) are now well established as potent anti‐cancer therapeutics due to their combination of specific targeting of tumor‐associated antigens and intracellular delivery of toxic payloads. Blockbuster successes such as Kadcyla and Enhertu—both of which target HER2—highlight the great potential of this class of therapeutics. However, direct cancer cell killing alone using ADCs is often not sufficient for durable responses due to the development of drug resistance [1]. Factors leading to drug resistance include insufficient tumor penetration, receptor downregulation, heterogeneous antigen expression, and loss of sensitivity to the payload [2, 3, 4, 5]. To further improve efficacy, combining ADCs with immunotherapy to overcome this resistance has shown promise [6]. For example, one of the most active areas of ADC polytherapy research is combining ADCs with checkpoint inhibitors to achieve even stronger therapeutic activities [7, 8]. The combination of enfortumab vedotin, an ADC specific for nectin‐4, and pembrolizumab, a checkpoint inhibitor specific for PD‐1, is approved for treating urothelial carcinoma [9], and topline results for sacituzumab govitecan (ADC specific for Trop2) and pembrolizumab show improvement in progression‐free survival, with overall survival data not yet mature [10]. Additionally, combinations of brentuximab vedotin (an ADC targeting CD30) with checkpoint inhibitors (nivolumab or pembrolizumab) are being evaluated in various cancers [11, 12].

Despite the promising results of such antibody/ADC mixtures, the benefit of checkpoint inhibitors is well known to be limited in cold tumors with few tumor‐infiltrating lymphocytes [13]. Thus, the combination of ADCs with checkpoint inhibitors is expected to only impact a subset of patients. A logical approach to address this limitation is to use agents that enhance innate immune responses, such as Toll‐Like Receptor (TLR) and STimulator of INterferon Genes (STING) agonists [14, 15, 16, 17]. Indeed, such agonists show strong anti‐cancer activity, including in cold tumors [18, 19], although they also suffer from strong side effects due to broad immune activation [20, 21]. To address this limitation, TLR and STING agonists have been conjugated to antibodies to generate immune‐stimulating antibody conjugates (ISACs) for specific targeting to tumor cells to locally induce immune activation [22]. For example, the systemic administration of an ISAC (TLR7/8 agonist conjugated to anti‐HER2 antibody) led to tumor inhibition and prolonged immunological memory by activating intra‐tumoral myeloid cells [23], while administration of a HER2‐directed STING agonist (XMT‐2056) induced an innate antitumor immune response upon systemic administration in mice [24]. Nevertheless, these agents can induce adverse effects at doses used for monotherapy, such as anti‐drug antibody (ADA) responses [22], cytokine release syndrome (CRS) [25], or rapid clearance [26].

One additional challenge related to ADCs—which need to be internalized into cancer cells to deliver their toxic payloads—is that some tumor‐associated antigens are slowly internalized, ultimately leading to insufficient payload delivery. While targets such as HER2 or nectin‐4 are rapidly internalized [27, 28], other attractive cancer targets—such as carcinoembryonic antigen (CEACAM5, which is herein referred to as CEA)—are internalized too slowly for efficient ADC delivery. For example, the slow internalization of CEA may have contributed to reduced efficacy with labetuzumab govitecan and tusamitamab ravtansine [29, 30, 31]. To improve the CEA internalization rate, the combination of non‐competitive CEA‐binding antibodies has been tested and shown to increase antibody internalization by cancer cells and improve efficacy [32]. Similar results have been observed with HER2 cross‐linking agents [33].

**Fig. 1:**
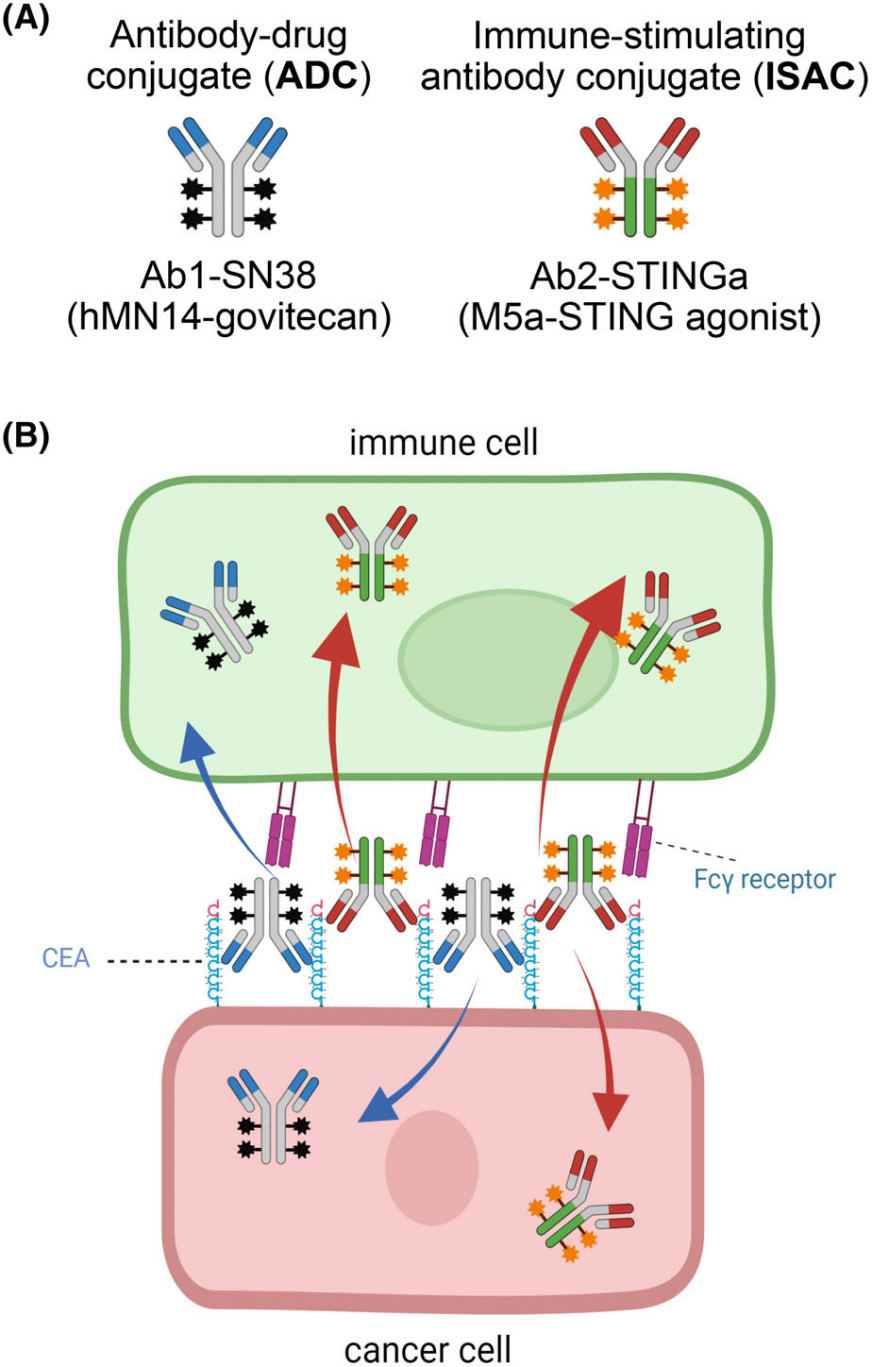
Overview of approach for combining antibody‐drug conjugates (ADCs) and immune‐stimulating antibody conjugates (ISACs) targeting carcinoembryonic antigen (CEA) to increase antitumor activity. (A, B) The two antibody conjugates in this study, namely (A) Ab1‐SN38 (hMN14‐govitecan) and Ab2‐STINGa (M5a‐STING agonist), are combined to achieve (B) internalization into both cancer cells to deliver the toxic payload (SN‐38) and immune cells to deliver the immune‐stimulating payload (STING agonist). The two antibodies are non‐competitive for CEA and enhance internalization into cancer cells. In addition, the antibody selection and Fc region of the ISAC is engineered to have higher affinity for FcγRs, leading to increased internalization into immune cells.

In this study, we have sought to develop a technology for increasing the efficacy of ADCs using mixtures of ADCs and ISACs with two key attributes (Fig. 1). First, to address the slow internalization of tumor‐associated antigens such as CEA, we have paired an anti‐CEA ADC with an ISAC that uses a second anti‐CEA antibody with a non‐competitive epitope to enhance internalization of the ADC into cancer cells. Second, to maximize the efficacy of the ISAC, we conjugated the STING agonist to the antibody that has more efficient engagement with Fcγ receptors (FcγRs) to improve internalization into immune cells, and we further engineered the Fc domain to increase Fc effector function. Herein, we report the generation and evaluation of the mixture of anti‐CEA ADC and ISAC agents and preclinical evaluation of their *in vivo* anti‐cancer activity.

## 2 Materials and methods

### 2.1 Cell lines

The HEK293‐6E cell line (RRID: CVCL_HF20) was purchased from National Research Council Canada and cultured in 50 mL disposable conical tubes with F17 (50591354; Thermo Fisher Scientific, Waltham, MA, USA) media with 4 mm L‐glutamine and 0.1% Kolliphor P188. CAPAN‐1 cell line (RRID: CVCL_0237) was purchased from ATCC and cultured in IMDM media (Fisher, Waltham, MA, USA, 12440053) with 20% HI FBS (Fisher; 10082147) and 1% P/S (Penicillin–Streptomycin; Fisher, 15140122). The J774A.1 cell line (RRID: CVCL_0358) was purchased from ATCC and cultured in DMEM media (Fisher, 11965092) with 10% HIFBS and 1% P/S. ADCC reporter line (RRID: CVCL_A7ZT) was purchased from Invivogen (jktl‐nfat‐cd16) and cultured in IMDM media with 10% HI FBS, 1% P/S, 100 μg·mL^−1^ Normocin (Invivogen, San Diego, CA, USA, ant‐nr‐1), 10 μg·mL^−1^ of Blasticidin (Invivogen, ant‐bl‐05), and 100 μg·mL^−1^ of Zeocin (Invivogen, ant‐zn‐05). The STING activation reporter cell line (IRF Reporter‐THP‐1; RRID: CVCL_F0PJ) was purchased from BPS Bioscience (79858) and cultured in RPMI1640 (Fisher, A1049101) with 10% HI FBS, 1% P/S, and 1 μg·mL^−1^ of Puromycin (Invivogen, ant‐pr‐1). LS174T (RRID: CVCL1384) was purchased from ATCC and cultured in EMEM media (ATCC, Manassas, VA, USA, 30–2003) with 10% HI FBS and 1% P/S. MC38‐CEA (RRID: CVCL_5I36) was purchased from Kerafast and cultured in DMEM (Fisher, 11960044) with 10% HIFBS, 1% P/S, 0.1 mm non‐essential amino acids (Fisher, 11140050), 1 mm sodium pyruvate (Fisher, 11360070), 2 mm Glutamine (Fisher, A2916801), 0.2 mm Gentamicin (Fisher, 15710064), 10 mm HEPES (Fisher, 15630106), and 300 μg·mL^−1^ G418(Fisher, 10131035).

All cells were purchased within the past 3 years from authenticated commercial sources. Once received, cells were aliquoted and frozen after a short propagation period. Aliquoted cells were maintained at 37 °C under a humid atmosphere containing 5% CO_2_, used for up to 60 days. All cells were routinely tested for mycoplasma, and all experiments were performed with mycoplasma‐free cells.

### 2.2 Cloning and recombinant antibody expression

For hMN14 (Ab1) and M5a (Ab2), VH and VL genes were ordered as gBlocks (IDT, Newark, NJ, USA). After PCR amplification, VH genes were digested with EcoRI‐HF (NEB, Ipswich, MA, USA, R3101L) and NheI‐HF (NEB, R3131L), and VL genes were digested with EcoRI‐HF and BsiWI‐HF (NEB, R3553L). Mammalian expression plasmids (pTT5) containing a common human IgG1 heavy or light chain (kappa) framework were digested with appropriate restriction enzymes and treated with calf intestinal alkaline phosphatase (CIP; NEB, M0525L). Digested inserts and vectors were analyzed by electrophoresis using a 1% agarose gel, purified by Qiagen Gel Extraction, ligated with T4 ligase (NEB, M0202L), and transformed by heat shock into DH5α E. coli cells. Sequences were confirmed by Sanger sequencing.

Enhanced Fc (eFc) fragments were ordered as gBlocks (IDT). The wild‐type Fc of human IgG1 was replaced with an enhanced Fc by overlap PCR. Five mutations (L235V, F243L, R292P, Y300L, and P396L) were introduced for eFc. The new heavy chains were digested with EcoRI‐HF (NEB, R3101L) and BamHI‐HF (R3136L) and inserted into pTT5 mammalian expression plasmids.

For antibody expression, soluble IgGs were produced by HEK293‐6E via transient transfection (30 mL) using 12.5 μg each of heavy and light chain plasmids mixed with 100 μg Polyethylenimine (PEI MAX, 247651; Polysciences Inc, Warrington, PA, USA). After transfection, 750 μL of 20% Yeastolate (Fisher, 255772) in DI water was added to each batch. Six days after transfection, cultures were harvested and centrifuged at 3500× ***g*** for 40 min. Supernatants were collected into 50 mL conical tubes.

For antibody purification, 0.5 mL of dry volume of Protein A beads (Thermo Fisher Scientific, 20333) per tube was added. Mixtures were gently shaken at 4 °C overnight. Protein A beads were then collected by vacuum filter columns (Thermo Fisher Scientific, 89898) and washed with ~100 mL of PBS. Antibodies were eluted using 1 mL of 0.1 m glycine buffer (pH 3.0), and then buffer exchanged to 0.1 m acetate (pH 5.0) with Zeba desalting columns (Thermo Fisher Scientific, 89890), and aliquoted and stored at −80 °C. The purity of antibodies was confirmed by SEC to ensure the purity is above 90%.

### 2.3 Antibody characterization

The purity of antibodies was tested by preparative size‐exclusion chromatography (SEC) using a Shimadzu Prominence semi‐prep HPLC System outfitted with a LC‐20AT pump, SIL‐20 AC autosampler, and FRC‐10A fraction collector. Proteins were loaded onto a SEC column (Superdex 200 Increase 10/300 GL column; GE, Boston, MA, USA, 28990944) and analyzed at 0.75 mL·min^−1^ using a PBS running buffer with 200 mm arginine (pH 7.4).

### 2.4 Antibody fluorescent labeling

Antibodies were concentrated to ~2 mg·mL^−1^ in 100 μL of PBS in 1.5 mL tubes. Sodium bicarbonate (10 μL of 7.5% solution; Millipore Sigma, Burlington, MA, USA, S6014) was added to each tube to adjust pH to ~8–8.5. Next, 2 μL of 10 mg·mL^−1^ Alexa‐647 NHS Ester (Fisher Scientific, A37573) was added to each tube. Tubes were put on a shaking plate for 1 h at room temperature. Next, Biogel (850 μL of 10% P6; Bio‐Rad, Hercules, CA, USA, 1504130) was added to a Costar‐Spin‐X tube (Corning, Corning, NY, USA, 07‐200‐387) and spun down at 3500× g for 1.5 min. The eluent from the bottom of the tube was removed, and then ~400 μL of 10% P6 Biogel was added. The tubes were spun down at 3500× g for 1.5 min. The eluent from the bottom of the tube was removed, and the reaction mixture was added to the top of the column. The tubes were spun down at 3500× g for 1.5 min. The eluent was collected into a new 1.5 mL tube. The protein concentration and degree of labeling were measured using a NanoDrop (Thermo, 840274100).

### 2.5 CEA receptor binding measurements

CAPAN‐1 cells in each T75 flask were treated with 3 mL of trypsin (Fisher, 25‐300‐054). After detaching, 6 mL of IMDM media was added, and the media was collected in 15 mL tubes. Cells were spun down at 500× g for 5 min and then rewashed and resuspended using PBS with 0.1% bovine serum albumin (PBSB). Antibodies were diluted in PBSB and added to 96‐well U‐bottom plates (Fisher, 2205) with 100 μL per well. Cells were added as 50 000 cells per well with 100 μL per well. Plates were then chilled on ice for 4 h. Afterward, cells were washed with PBSB at 500× g for 5 min and then resuspended in 100 μL PBSB. Alexa anti‐human Fc (Jackson ImmunoResearch, West Grove, PA, USA, 109‐605‐190) was diluted with PBSB 300‐fold and added to each well as 100 μL. Plates were incubated on ice for 4 min. Then cells were washed with PBSB at 500× g for 5 min and then resuspended in 100 μL PBSB. Fluorescent signals were measured by flow cytometry (Bio‐Rad Ze5).

### 2.6 Antibody competitive binding measurements

CAPAN‐1 cells were cultured as discussed above. After detachment, the cells were added as 50 000 cells per well in PBSB at a total volume of 100 μL per well and then chilled on ice. hMN14 (Ab1) or M5a (Ab2) IgGs (100 nm) were added to the cells and then incubated on ice (1 h). Fluorescently labeled M5a (Ab2) (10 nm) was added to the cells and incubated on ice (4 h). Afterward, the cells were washed with PBSB at 500× g for 5 min and then resuspended in 100 μL of PBSB. Fluorescent signals were measured by flow cytometry.

### 2.7 Antibody‐dependent cell‐mediated cytotoxicity (ADCC) assays

The ADCC assay was modified from the manufacturer's protocol. CAPAN‐1 cells were seeded into 96‐well Tc‐treated flat‐bottom plates (Corning, 3585) as 50 000 cells per well and incubated at 37 °C and 5% CO_2_ overnight. On the second day, antibodies were added alone or as mixtures at different concentrations. Next, 110 μL of the diluted antibody was added to each well and incubated at 37 °C and 5% CO_2_ for 1 h. The ADCC reporter cells were then centrifuged at 300× ***g*** for 5 min and resuspended in the test medium (IMDM media with 10% HI FBS, 1% P/S). Ninety microliters of ADCC reporter cells (0.2 million cells) per well were added to the cancer cells, and the plates were incubated at 37 °C and 5% CO_2_ for 6 h. After incubation, 20 μL of the supernatant was transferred into a white assay plate (Fisher, 720091). Finally, 50 μL of QUANTI‐Luc^TM^ (Invivogen, REPQLC2) was added per well, and the luminescence values were measured immediately. Raw signals were normalized by the signal for the negative control, which was the mixture of cancer cells and reporter cells without antibodies.

### 2.8 Antibody co‐culture internalization assays

Celltrace Violet (Fisher, 34557) and Far Red (Fisher, 34564) staining solution were dissolved in 20 μL of DMSO, and 10 μL solutions of Cell Trace violet or Far Red were added to 10 mL of PBS in 15 mL tubes. Next, the media was removed, and the cells were washed with 10 mL of PBS. Far Red staining solution was added to J774A.1 cells, and violet staining solution was added to CAPAN‐1 cells. Both types of cells were separately incubated at 37 °C and 5% CO_2_ for 20 min, and then the staining process was quenched by adding 10 mL of complete medium. Cells were then seeded into 96‐well Tc‐treated flat‐bottom plates (Corning, 3585) as 40 000 cells per well with different macrophage:cancer ratios and incubated at 37 °C and 5% CO_2_ overnight. On the second day, medium was aspirated and 100 μL of fresh medium was added with 40 nm of AF‐488 (Fisher, A20000) fluorescently labeled Ab2 (M5a) or Ab2* (M5a‐eFc) antibodies, including with or without equimolar concentrations of Ab1 (hMN14). Cells were incubated at 37 °C and 5% CO_2_ for 4 h and then detached. Fluorescent signals with or without trypan blue quenching were measured by flow cytometer. Internalized antibodies were represented by fluorescent signals after quenching.

### 2.9 Antibody STING agonist conjugation

Conjugatable STING ligand (Invivogen, vac‐stg982), a conjugatable ligand engineered from *CL845*, which is an analog of the clinical STING agonist CL656, was applied to make the antibody STING agonist conjugate [34, 35]. The conjugation process was performed according to the manufacturer's instructions. Briefly, the antibody was prepared at ~5 mg·mL^−1^ in conjugation buffer, which includes 0.1 m sodium phosphate, 0.1 m NaCl, and 1 mm EDTA (pH 7.5). Next, 1 mm of TCEP (tris(2‐carboxyethyl) phosphine; Millipore Sigma, C4706) was added to reduce disulfide bonds, followed by incubation at 37 °C for 2 h. STG‐982 (0.25 mg) was dissolved in 30 μL of sterile DMSO and then mixed with reduced mAb (Ab2*) at a STG‐982/mAb molar ratio of 3 or 8; mixtures were incubated at 4 °C for 4 h, and then desalted against a quenching buffer (0.1 m disodium phosphate, 0.1 m NaCl, 50 mm sodium tetraborax, pH 8.0) and stored in final buffer (20 mm sodium phosphate, 0.15 m NaCl, pH 6.5–7.5). The DARs of STING agonist conjugates were confirmed by UV–vis. The conjugates were then filtered and stored at −20 °C.

### 2.10 DAR quantification

The STING ligand (drug) to antibody ratio (DAR) was evaluated by first measuring the ratio of OD260nm to OD280nm of the antibody, conjugatable STING agonist, and desalted antibody conjugate via nanodrop and then calculating the DAR per the manufacturer's instructions.

### 2.11 STING agonist *in vitro* activation assay

CAPAN‐1 cells were seeded at 40 000 cells per well into a white, clear‐bottom 96‐well microplate (Fisher, 2205) in 50 μL of assay medium. STING activation reporter cells (25 μL) were added at 40 000 cells per well into wells with CAPAN‐1 cells (50 μL) for a total volume of 75 μL. Next, antibody conjugates at different concentrations were added to the wells (25 μL) to make a final volume of 100 μL. Samples were incubated at 37 °C with 5% CO_2_ for 24 h. On the second day, luciferase assay reagent (100 μL) was added to each well and stored at room temperature for 30 min. Luminescence signals were measured immediately using a luminometer.

### 2.12 STING agonist *in vitro* toxicity assay

CAPAN‐1 cells were seeded at 5000 cells per well in 100 μL of culture medium and incubated at 37 °C and 5% CO_2_ overnight. On the second day, antibodies or STING agonist conjugates were diluted, and 100 μL of the diluted antibodies or conjugates were added to each well and incubated at 37 °C and 5% CO_2_. On Day 4, the number of viable cells in culture was measured using a Cell Titer‐Glo Luminescent Cell Viability Assay kit (Promega, Madison, WI, USA, G7571).

### 2.13 ADC (hMN14‐SN38) *in vitro* toxicity assay

Cancer cells were seeded at 5000 cells per well in 100 μL of culture medium and incubated at 37 °C and 5% CO_2_ overnight. On the second day, hMN14‐SN38 (MedChemExpress, Monmouth Junction, NJ, USA, HY‐P9968) was serially diluted, and 100 μL of the diluted hMN14‐SN38 was added to each well and incubated at 37 °C and 5% CO_2_. On Day 4, the number of viable cells in culture was measured using a Cell Titer‐Glo Luminescent Cell Viability Assay kit (Promega, G7571).

### 2.14 Animal studies

The study was performed in accordance with and approval from the University of Michigan Institutional Animal Care and Use Committee (PHS (Washington, D.C., USA) Assurance Number D16‐00072 (A3114‐01)) and Association for Assessment and Accreditation of Laboratory Animal Care International guidelines (Accreditation Unit Number 000285). Female Nu/J (002019; *Foxn1* ^*nu*^) mice, aged 6–8 weeks, were used in both the efficacy and histology studies. *Foxn1* ^*nu*^ (nude) mice are hairless and athymic, resulting in a lack of T cells and a lack of cell‐mediated immunity. The efficacy study involved 10 mice per group (70 in total). The histology study involved three mice per group (12 in total).

All housing and experimental procedures on mice were performed in a safety hood in a dedicated mouse room, as *Foxn1* ^*nu*^ mice are susceptible to C. bovis infection. Physiological acclimatization was applied to mice for 7 days after delivery to a C‐bovis‐free mouse room (4 mice per cage). The husbandry and housing were performed by the Unit for Laboratory Animal Medicine (ULAM) at the University of Michigan. Mice were randomly assigned to cages before tumor inoculation, and the cages were placed randomly on the shelf. The same researcher performed all injections and measurements to minimize potential confounding factors. When inoculating the tumor and injecting the drug, mice were anesthetized with isoflurane. No blinding was conducted.

The outcomes of efficacy studies are the treatment efficacy and potential side effects, as represented by changes in tumor volume and body weight. The outcome of the histology studies is how the ISAC contributes to tumor inhibition, which is represented by the percentage of tumor infiltration area.

### 2.15 *In vivo* efficacy study

All animal studies were conducted in accordance with the University of Michigan Institutional Animal Care and Use Committee. For the efficacy study, *Foxn1* ^*nu*^ mice (Jackson Labs, Bar Harbor, ME, USA, 002019) were injected with 5 million cells of CAPAN‐1 cells into the left flank with 50% v/v Matrigel. Tumor volume was measured using calipers and the formula of length^2^ × width/2, where length is the shorter dimension of the tumor. Once tumors reached a volume of approximately 250 mm^3^, mice were administered one of the following treatments via tail vein injection: (i) PBS vehicle control; (ii) 10 mg·kg^−1^ M5a‐eFc1 (Ab2*); (iii) 10 mg·kg^−1^ hMN14‐SN38 (ADC); (iv) 10 mg·kg^−1^ M5a‐eFc1 (Ab2*) and 10 mg·kg^−1^ hMN14‐SN38 (ADC); (v) 10 mg·kg^−1^ M5a‐eFc1‐STING agonist (ISAC); (vi) 10 mg·kg^−1^ M5a‐eFc1‐STING (ISAC) and 10 mg·kg^−1^ hMN14 (Ab1); or (vii) 10 mg·kg^−1^ M5a‐eFc1‐STING (ISAC) agonist and 10 mg·kg^−1^ hMN14‐SN38 (ADC). Eight mice were tested in the control (PBS) group, and 10 mice were tested in each treatment group. The drug‐to‐antibody ratios were constant (DAR6 for ISAC and DAR8 for ADC), and because the antibody molecular weight was so much larger than the payload, we did not adjust for the increased molecular weight (~6% difference) of payload conjugation. The hMN14 (Ab1) alone group was excluded due to its low efficacy [36]. On day 7 (post‐first injection), mice were administered the same treatment with the same dosage for a second injection. Body weights and tumor sizes were monitored twice weekly until tumors reached the humane endpoint.

### 2.16 Histology imaging

*Foxn1* ^*nu*^ mice (Jackson Labs, 002019) were injected with 5 million CAPAN‐1 cells into the left flank with 50% v/v Matrigel. Once tumors reached a volume of approximately 250 mm^3^, mice were administered one of the following treatments via tail vein injection: (i) PBS vehicle control; (ii) 10 mg·kg^−1^ hMN14‐SN38 (ADC); (iii) 10 mg·kg^−1^ M5a‐eFc1‐STING agonist (ISAC); and (iv) 10 mg·kg^−1^ M5a‐eFc1‐STING (ISAC) agonist and 10 mg·kg^−1^ hMN14‐SN38 (ADC). Tumors were harvested after 7 days of injection and frozen at −80 °C. Frozen tumors for histology were sectioned using a cryostat into 10 μm slices. Sections were stained with anti‐mouse CD45‐FITC (BioLegend, San Diego, CA, USA, 103107) and AF647‐labeled trastuzumab for 30 min in PBS‐BSA, and then stained with Hoechst (Invitrogen, 33342) for 5 min in PBS‐BSA. Sections were then washed with PBS before imaging. Microscopy was performed using a 20× objective on an Olympus FV1200 microscope using 405 nm, 488 nm, and 635 nm lasers, and images were analyzed in ImageJ.

### 2.17 Statistics

All statistical analyses were performed using graphpad prism. For comparison of tumor volume, weight change, and CD45^+^ area, an unpaired *t*‐test was used. Kaplan–Meier survival curves were plotted based on the humane endpoint (tumor volume 2000 mm^3^). A *p*‐value less than 0.05 is considered statistically significant.

## 3 Results

### 3.1 Competition analysis of antibodies targeting CEA

**Fig. 2:**
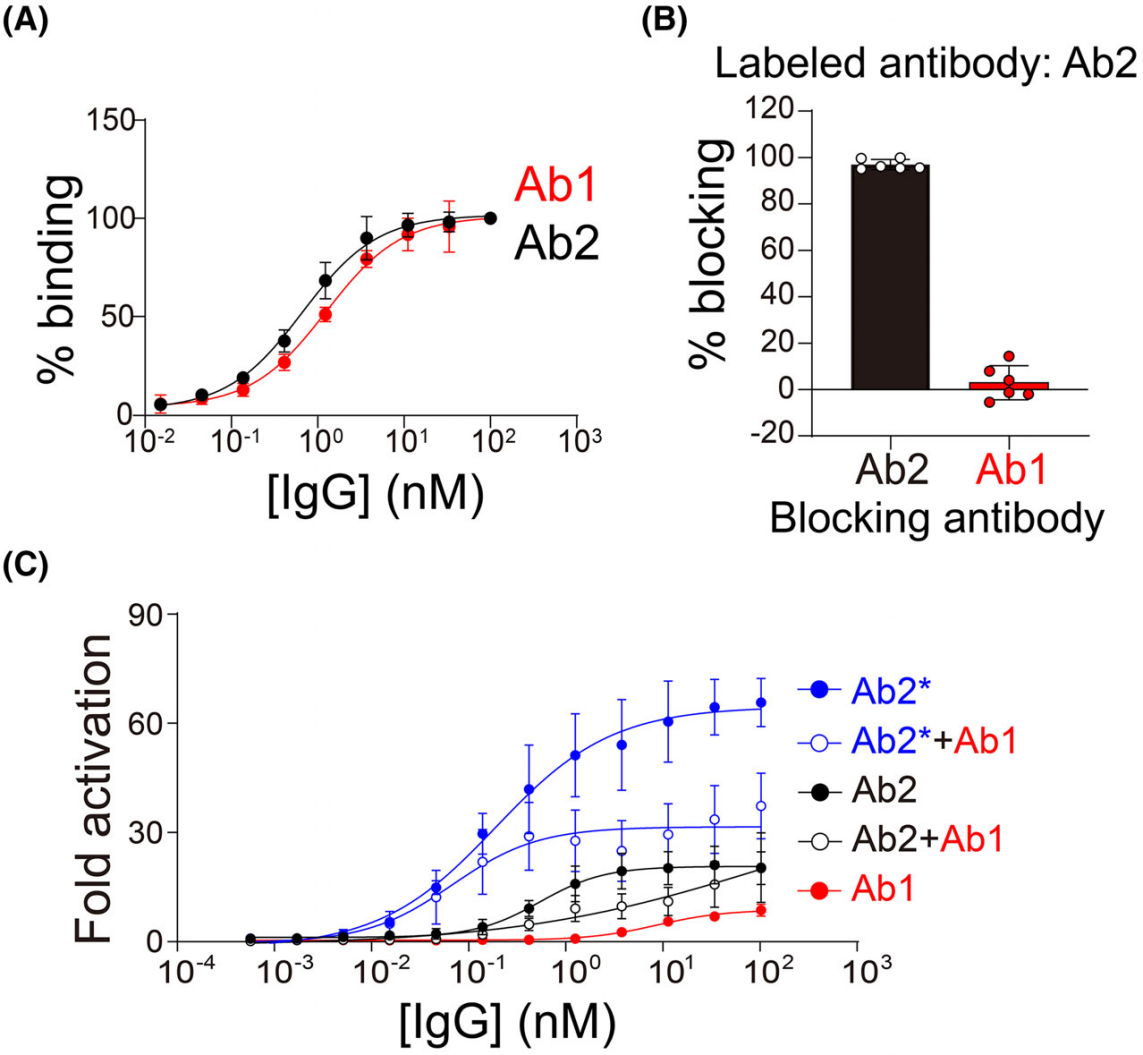
Mixtures of non‐competitive anti‐CEA antibodies activate antibody‐dependent cellular cytotoxicity (ADCC) reporter cells. (A) Flow cytometry binding curves of Ab1 and Ab2 binding to a CEA‐expressing cell line (CAPAN‐1) with secondary antibody detection. (B) The percentage of antibody blocking, as judged by the binding of fluorescently labeled Ab2 (Ab2‐AF647; 10 nm) after pre‐blocking with unlabeled Ab1 or Ab2 (100 nm). (C) ADCC reporter assay for individual antibodies or mixtures thereof using CAPAN‐1 cells. Ab2 is M5a (wild‐type Fc) and Ab2* is M5a with a mutated immune‐enhancing Fc region. The reported concentrations for the mixtures are not total antibody concentrations but rather the concentrations of each antibody component (i.e., a mixture of 1 nm Ab1 and 1 nm Ab2 is reported as 1 nm). The signal of the cancer cells and reporter cells mixture without antibodies was used as control. In (A–C), the data are averages of three independent experiments and the error bars are standard deviations.

Two antibodies were selected for the development of the ADC and ISAC agents, namely labetuzumab (Ab1), which binds domain A3B3 of CEACAM5 [37, 38], and M5A (Ab2), a humanized version of T84.66 that binds domain A3 [39, 40]. Toward our goal of utilizing two IgGs targeting the same receptor, we first evaluated their potential competition with each other in terms of binding CEA and activating FcγRs (Fig. 2). Thus, we evaluated the binding of Ab1 and Ab2 to CEA receptors using the CAPAN‐1 cell line, a pancreatic cancer cell line with high expression of CEA (230 000 receptors per cell) [41] (Fig. 2A). Both IgGs showed similar binding affinity (Ab1: EC_50_ = 1.26 nm, Ab2: EC_50_ = 0.67 nm) and saturated CEA receptors at around 10 nm. Next, we performed a blocking assay using 10 nm of fluorescently labeled Ab2 in the presence of a 10‐fold excess (100 nm) of unlabeled Ab1 or Ab2 (Fig. 2B). As expected, unlabeled Ab2 substantially reduced the binding of labeled Ab2. In contrast, the presence of unlabeled Ab1 had a minimal effect on Ab2 binding, indicating that Ab1 and Ab2 can bind to CEA receptors without competing with each other on the cancer cell surface.

We next evaluated the ability of the antibodies to activate FcγRs using an antibody‐dependent cellular cytotoxicity (ADCC) reporter cell assay (Fig. 2C). Our motivation for these studies is that the ISAC is expected to exert dual immunomodulatory effects, including being internalized into immune cells to activate the STING pathway and forming crosslinks between cancer and immune cells to induce ADCC and antibody‐dependent cellular phagocytosis (ADCP). Both mechanisms are dependent on the engagement of FcγRs. While the ADC can also exert Fc effector functions, its primary role is internalization into the cancer cells. Therefore, we tested the engagement of both antibodies with Fc receptors to select the optimal choice for the IgGs of the ISAC and ADC. As single agents, Ab2 was more potent (i.e., lower EC_50_) and active (i.e., higher maximum fold activation) than Ab1 in the ADCC assay.

As both Ab1 and Ab2 possess a human IgG1 framework with effector‐competent Fc regions, we next evaluated whether Ab1 interferes with Ab2 when forming crosslinks between cancer and immune cells by an ADCC reporter cell assay (Fig. 2C). Ab2 alone showed moderate activity (20‐fold relative to untreated control, IC_50_ = 0.53 nm), while Ab1 showed lower activity (~10‐fold relative to untreated control, IC_50_ = 8.55 nm). The low Fc effector function of Ab1 (hMN14) is consistent with a previous study, which showed hMN14 (Ab1) had negligible efficacy alone [36]. Interestingly, equimolar (1:1) mixtures of Ab1 and Ab2 resulted in intermediate activity, indicating some interference with cross‐linking in the immune synapse between the cancer and ADCC reporter cell lines. To improve the immune response of Ab2, five mutations in the Fc domain (L235V, F243L, R292P, Y300L, and P396L) [42, 43, 44] were introduced to make an immune‐enhancing version of Ab2 (Ab2*), which showed an increased activity of 60‐fold relative to control and an IC_50_ of 0.19 nm. When Ab2* was mixed with Ab1 at a 1:1 ratio, the activity was ~30‐fold relative to untreated control (IC_50_ of 0.12 nm). Although the maximum efficacy is reduced compared to Ab2* alone, the potency of the mixture still exceeded Ab2 alone (Fig. 2C). Therefore, Ab2* is suitable for engaging FcγRs even in combination with Ab1.

### 3.2 Comparison of internalization of antibodies targeting CEA into cancer and macrophage cells

**Fig. 3:**
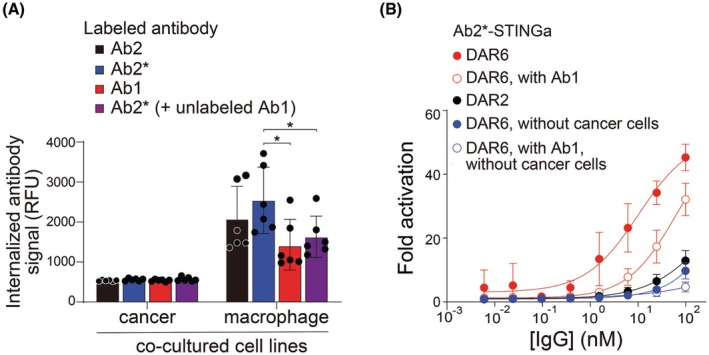
Anti‐CEA antibodies display unique propensities to be internalized into macrophages and deliver a STING agonist when co‐cultured with cancer cells. (A) Cancer (CAPAN‐1) and macrophage (J774A.1) cells were mixed (9 : 1 ratio), seeded in 96‐well plates, and fluorescently labeled IgGs (40 nm each) were added at 37 °C for 4 h. Next, the internalized fluorescent signals were evaluated using flow cytometry (see Methods for more details). Ab2* is Ab2 with a mutated immune‐enhancing Fc. The data are averages of three independent experiments and the error bars are standard deviations. The statistical significance was evaluated using an unpaired *t*‐test and *p*‐values of < 0.05 (*) are indicated on the plots. (B) The ISAC Ab2*‐STINGa was incubated with co‐cultures of cancer (CAPAN‐1) and reporter (THP‐1 IRF) cells for 24 h (37 °C), and the luciferase assay reagent was added to samples, and the luminescence signal was quantified. The reported concentrations for the mixtures (equimolar amounts) are not total antibody concentrations but rather the concentrations of each antibody component. Ab2* is Ab2 with a mutated immune‐enhancing Fc. The data are averages of three independent experiments, and the error bars are standard deviations.

We next performed a co‐culture assay to investigate the cellular internalization behavior of the anti‐CEA antibodies into both cancer cells and macrophages (Fig. 3A). Ab2 and Ab2* showed more internalization into macrophages compared to Ab1, indicating that the former antibodies (Ab2 and Ab2*) are more suitable to deliver STING agonists into immune cells while the latter antibody (Ab1) is more suitable for delivering the toxic drug (i.e., SN38) into cancer cells (due to lower immune cell uptake). Fortuitously, the optimal ISAC properties of Ab2* resulted in Ab1 for the ADC, corresponding to a clinically tested compound (labetuzumab govitecan, Ab1‐SN38). This ADC is well‐tolerated in the clinic even at high doses (e.g., weekly doses of 10 mg·kg^−1^) [30].

To further test the macrophage internalization in mixtures of Ab1 (to deliver toxic drug to cancer cells) and Ab2* (to deliver an immune agonist to immune cells), we conducted additional co‐culture assays with antibody mixtures (Fig. 3A). When mixed with Ab1, the internalization of Ab2* into macrophages is reduced due to the Ab1 Fc blocking effect, which is consistent with the ADCC reporter cell assay results (Fig. 2C). To characterize the antibody uptake, we altered the cancer:macrophage cell ratio in co‐culture and evaluated internalization into each cell type (Fig. S1A,B). The antibodies showed similar internalization into cancer cells, which is low for CEA over the 4‐h incubation time. However, macrophage uptake was increased as the cancer‐to‐macrophage cell ratio was increased, suggesting enhanced immune synapse formation when individual macrophages engaged multiple cancer cells. To ensure the macrophage cells would not prevent the internalization of the cytotoxic ADC into cancer cells, we measured the Ab1 internalization into CAPAN‐1 cells with an excess (9 : 1 ratio) of J774A.1 cells. While there was a small (but statistically significant) reduction in the cell surface signal of Ab1, the cancer cell internalized antibody signal did not change (Fig. S1C).

### 3.3 Preparation of functional ISACs with improved tumor specificity

To generate functional ISACs with STING agonist activity, we prepared Ab2*‐STINGa (ISAC) conjugates with distinct drug‐to‐antibody ratios (DARs), namely DAR2 (two drug molecules conjugated to each antibody molecule) and DAR6. Their STING activation effect was evaluated through an IRF Reporter‐THP‐1 cell assay when co‐cultured with CAPAN‐1 cells as the cancer cell line. The ISACs – in the absence of a second antibody – showed a maximum of 12.8‐fold (DAR2) and ~45.3‐fold (DAR6) activation relative to control (Fig. 3B). Based on the higher activity, DAR6 was selected for further analysis. Next, we evaluated the impact of conjugation on binding affinity. Previous research has demonstrated equivalent affinity of hMN14 (Ab1) and labetuzumab govitecan (Ab1‐SN38) [45] for the cytotoxic ADC, and we measured the affinity of Ab2* and Ab2*‐STINGa to CEA receptors with CAPAN‐1 cell line to show the same for the ISAC. The similar affinity of Ab2* (EC_50_ of 0.78 nm) and Ab2*‐STINGa (EC_50_ of 0.71 nm) indicated the affinity is not affected by the conjugation (Fig. S2).

To evaluate selectivity, DAR6 Ab2*‐STINGa (ISAC) activation was also measured without target cells, resulting in lower maximum levels of activation (9.6‐fold). This achieved a selectivity of 4.7, as defined by the activity with cancer cells versus without cancer cells. Next, the DAR6 Ab2*‐STINGa (ISAC) was mixed with an equimolar ratio of Ab1. A maximum of 32.1‐fold activation of the reporter cell line was observed in the presence of cancer cells versus a 4.5‐fold activation without cancer cells, resulting in an increased selectivity (i.e., 7.1‐fold relative to control without cancer cells). The increase in selectivity could occur from the preferential FcγR binding for Ab2* relative to Ab1 in the synapse between the cancer and reporter cells because the 1:1 mixture has less than the expected reduction of activity (i.e., < 50% reduction) in the absence of the synapse, as observed for the 1:1 mixture of Ab2*‐STINGa (ISAC) and Ab1 (antibody without cytotoxic payload) without cancer cells. This *in vitro* test showed that the antibody combination could reduce uptake in the absence of cancer cells while maintaining most of its activity in the presence of cancer cells.

STING agonists can sometimes be toxic to cancer cells [46, 47], which led us to test the toxicity of Ab2*‐STINGa (ISAC) in cell culture using the CAPAN‐1 cell line (Fig. S3). However, no apparent toxicity was observed over a wide range of ISAC concentrations (0.01–100 nm).

### 3.4 Combining ADC and ISAC results in better *in vivo* tumor inhibition and increased immune response

**Fig. 4:**
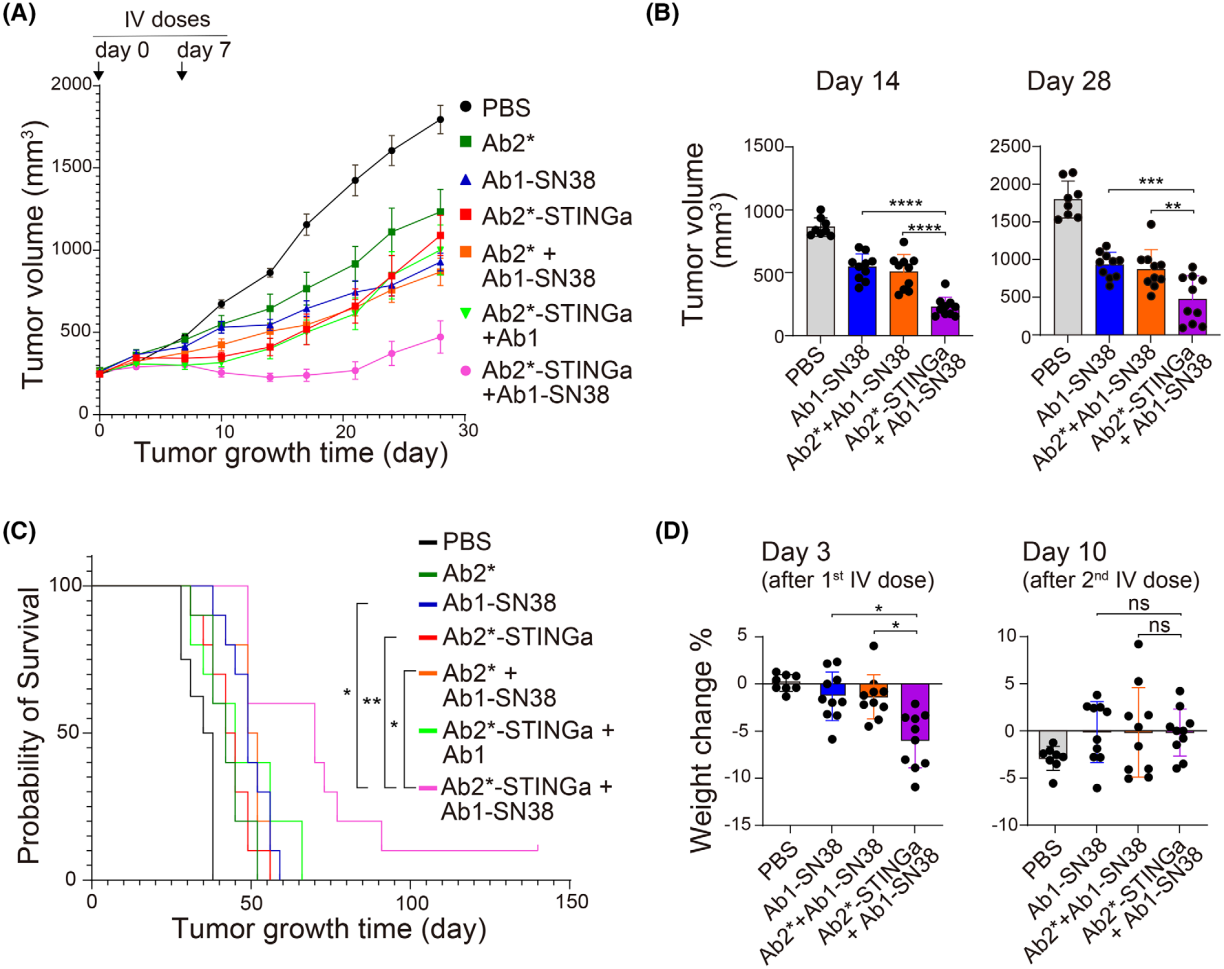
ADC and ISAC mixture displays the strongest anti‐cancer activity *in vivo*. (A–D) CAPAN‐1 cells were injected into immunodeficient (*Foxn1* ^*nu*^) mice, tumor volumes were allowed to reach ~250 mm^3^ (denoted as day 0), and then antibodies (10 mg·kg^−1^ each resulting in a total of 20 mg·kg^−1^ for mixtures) were administered twice (days 0 and 7) via tail vein injection. The tumor volumes were recorded twice per week. (A) The tumor volumes (mm^3^) versus time after various treatments. (B) Statistical analysis of tumor volumes on days 14 and 28. (C) Survival curves after various treatments. (D) Statistical analysis of weight loss after the first and second intravenous doses. Ab2* is Ab2 with a mutated immune‐enhancing Fc. The values are averages (*n* = 10 mice per group), and error bars are standard errors. The statistical significance was evaluated using an unpaired *t*‐test and *p*‐values of > 0.05 (ns), < 0.05 (*), < 0.01(**), < 0.001 (***), and < 0.0001 (****) are indicated on the plots.

Before conducting *in vivo* efficacy studies, we quantified the expression level of CEA on different cell lines. Even though the CEA expression on a mouse cancer cell line (MC38‐CEA) was much higher than that on human cancer cells, the Ab1‐SN38 (ADC) efficacy on human cancer cells is higher than that on mouse cells, consistent with the lower sensitivity of mouse cancer cell lines to many ADC payloads (Fig. S4). We therefore tested the *in vivo* efficacy of the single‐agent therapeutics and combinations of the ADC and ISAC with human CAPAN‐1 cells in immunodeficient mice (*Foxn1* ^*nu*^) (Fig. 4). We chose a STING agonist with moderate potency (versus more potent compounds) with the goal of increasing tolerability to enable dosing a high level of ISAC (e.g., 10 mg·kg^−1^) to aid in CEA cross‐linking, although ultimately more advanced preclinical and clinical tests are needed to establish tolerability. The dose of ISAC was determined by a pilot experiment with doses of 1, 3, and 9 mg·kg^−1^. Only the 9 mg·kg^−1^ showed significant changes in tumor growth, consistent with the moderate potency, and 10 mg·kg^−1^ was selected for the efficacy studies.

In CAPAN‐1 cancer xenografts, two doses (days 0 and 7) of Ab1‐SN38 (ADC), Ab2*‐STINGa (ISAC), and their combination were administered by tail vein injection, and the tumor growth rates were evaluated (Fig. 4A). The combination of Ab1‐SN38 (ADC) and Ab2*‐STINGa (ISAC) provided the strongest efficacy, and it was the only cohort in which a reduction of tumor volume was observed. Notably, the Ab1‐SN38 (ADC) + Ab2*‐STINGa (ISAC) combination achieved the highest tumor inhibition on day 14 after treatment initiation (median tumor size 218.7 mm^3^, CI 95%: 178.2–274.4 mm^3^), showing statistical significance compared to Ab1‐SN38 (ADC) (median tumor size 561.2 mm^3^, CI 95%: 481.2–610.5 mm^3^), or Ab1‐SN38 (ADC) + Ab2* (ISAC backbone) groups (median tumor size 549.8 mm^3^, CI 95%: 422.9–592.0 mm^3^) (Fig. 4B). This difference was maintained to the end of the experiment on day 28, at which time point Ab1‐SN38 (ADC) + Ab2*‐STINGa (ISAC) group still showed a significantly smaller tumor size (median tumor size 457.7 mm^3^, CI 95%: 278.4–666.6 mm^3^) compared to Ab1‐SN38 (ADC) only (median tumor size 971.8 mm^3^, CI 95%: 823.2–1032.3 mm^3^) or Ab1‐SN38 (ADC) + Ab2* (ISAC backbone) groups (median tumor size 836.5 mm^3^, CI 95%: 704.5–1031.3 mm^3^). The nude mouse model (*Foxn1* ^*nu*^) used in the study is immunodeficient and cannot form a lasting adaptive immune response. Likewise, there can be variability between mice in the extent of immune response as seen with individual growth curves (Fig. S5). However, the Ab1‐SN38 (ADC) + Ab2*‐STINGa (ISAC) group still showed significantly longer survival time based on Kaplan–Meier survival curves (Fig. 4C).

Comparisons among cohorts indicate that multiple mechanisms of action contribute to efficacy (Fig. 4A). The Ab2* (ISAC antibody backbone) alone group had some growth inhibition relative to the control, likely from the enhanced Fc effector function. The SN38 payload was also critical, as seen with the monotherapy result with Ab1‐SN38 (ADC) and the significant increase in efficacy between Ab2*‐STINGa (ISAC) co‐administered with either Ab1 alone or Ab1‐SN38 (ADC). Finally, the STING agonist is important given the difference between Ab1‐SN38 (ADC) co‐administered with either Ab2* (ISAC antibody backbone) or Ab2*‐STINGa (ISAC).

The lack of difference between some cohorts is also informative (Fig. 4A). Similar efficacy was seen between Ab2*‐STINGa (ISAC) alone and co‐administered with Ab1, indicating that the competitive binding to the cancer cell surface and changes in internalization did not interfere with the efficacy of Ab2*‐STINGa (ISAC). Notably, the addition of the cross‐linking antibody to increase ADC uptake (Ab2* + Ab1‐SN38) was numerically only slightly better than Ab1‐SN38 alone. This is in contrast to previous results showing an increase in efficacy when cross‐linking receptors with labetuzumab govitecan [32], although this could be due to the fewer doses and increased interval between ADC doses in the current study and/or different animal models.

We also measured the percentage of weight loss after treatment to evaluate the side effects (Figs 4D and S6). The Ab1‐SN38 (ADC) monotherapy group showed minor weight loss after treatment (median weight change −1.76%, CI 95%: −2.89% to 0.30%), while all groups that included the STING agonist (Ab2*‐STINGa) showed ~7% weight loss after the first  treatment. Ab1‐SN38 (ADC) + Ab2*‐STINGa (ISAC) combination has a median weight change of −5.38% with CI 95%: −7.78% to −4.15%, which was statistically significantly larger than for the Ab1‐SN38 (ADC) group. However, after the second treatment, the weight loss in the Ab2*‐STINGa (ISAC) + Ab1‐SN38 (ADC) group was minor (median: 0, CI 95%: −1.71% to 1.38%) and not different compared to the Ab1‐SN38 (ADC) group (median: 0.56, CI 95%: −2.11% to 1.90%), indicating that mice had adjusted to immune stimulation.

**Fig. 5:**
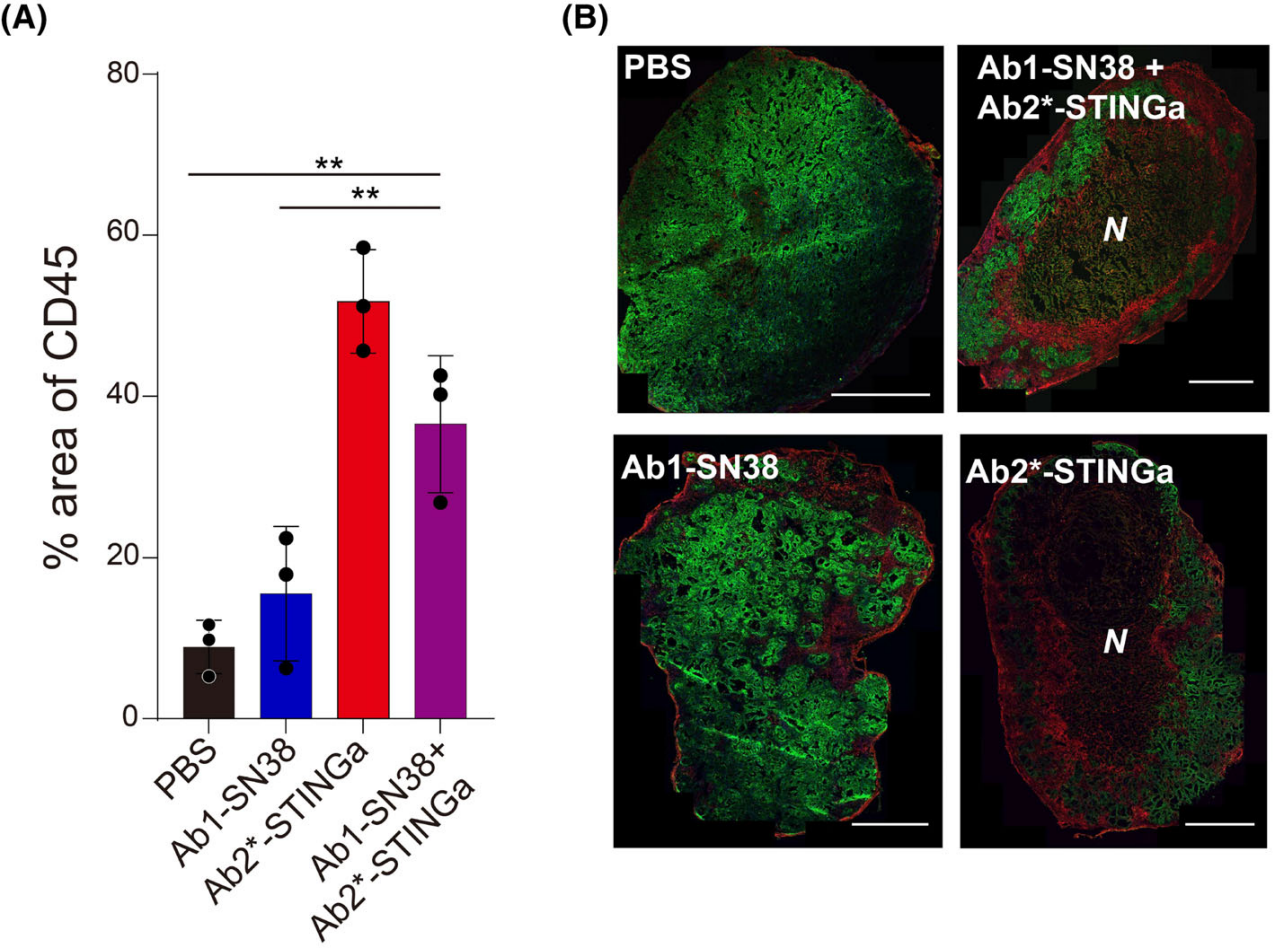
ISACs mediate infiltration of immune cells into tumors when administered alone or in combination with ADCs. (A,B) The tumors were generated as described in Fig. 4, resected after 7 days post administration of a single treatment on day 0, and sectioned. Tumor slides were stained and imaged using confocal microscopy. (A) The percentage of CD45‐positive area in tumors. (B) Representative images of tumors with different treatments. CD45 (red, immune cells), HER2 (green, expressed on cancer cells in addition to CEA), and Hoechst (blue) were imaged, and the scale bars are 1 mm. *N* stands for necrosis in the tumor, and this area was not included in the analysis in (A). Ab2* is Ab2 with a mutated immune‐enhancing Fc. The data are averages for three tumors, and the error bars are standard deviations. The statistical significance was evaluated using an unpaired *t*‐test and *p*‐values of < 0.01 (**) are indicated on the plots.

To observe the mechanisms behind these responses, we next measured the infiltration of CD45^+^ cells *in vivo* with different treatments to examine how Ab2*‐STINGa (ISAC) inhibits tumor growth by activating the immune system (Fig. 5). Responses to ISACs can start occurring within a day [48], while ADCs may take longer for their full effect [49, 50]. Therefore, we decided to analyze the tumors on day 7, prior to the second dose. The percentage of CD45^+^ area in the histology slides of tumors for the PBS‐treated group was ~10%. In the Ab1‐SN38 (ADC) group, the CD45^+^ area slightly increased to ~15%, consistent with Ab1‐SN38 (ADC) inhibiting tumor growth mainly through cancer cell toxicity. In the Ab2*‐STINGa (ISAC) and Ab1‐SN38 (ADC) + Ab2*‐STINGa (ISAC) groups, however, the CD45^+^ area reached ~50% and ~38%, respectively, both of which are significantly higher than those of the PBS or Ab1‐SN38 (ADC) groups. These results demonstrate differential immune cell infiltration, consistent with an Ab2*‐STINGa mechanism of action of stimulating an immune response to inhibit tumor growth (Fig. 5A). The Ab1‐SN38 (ADC) + Ab2*‐STINGa (ISAC) group had a smaller CD45^+^ area compared to the ISAC‐only group (*p* = 0.068). This could occur either from the Ab1 (ADC) Fc domain competing with Ab2*‐STINGa (ISAC) uptake or the payload effects of the ADC on the immune cells. Central tumor necrosis with a larger number of CD45^+^ cells around the necrotic area was a notable feature observed in the Ab2*‐STINGa (ISAC)‐treated groups (Fig. 5B), consistent with observations for other STING agonists [51]. Among the four different treatment groups, necrosis was commonly observed in groups with Ab2*‐STINGa (ISAC), while only one tumor treated with PBS showed such necrosis (Fig. S7).

## 4 Discussion

ADCs have made dramatic progress in the clinic for solid tumors, with 13 FDA‐approved agents [52]. However, resistance commonly develops, and durable antitumor responses often require a strong immune component. In many ways, ADCs are ideal agents for this by selectively delivering a cytotoxic agent to cancer cells and eliciting immunogenic cell death. Unlike chemotherapy, which can also elicit immunogenic cell death, ADCs avoid large uptake into immune cells and the associated immunosuppression that occurs with classic chemotherapy [53]. In addition to the selective cell killing, the Fc domain of an ADC can directly stimulate immune responses such as ADCC and ADCP and exert a vaccinal effect through mechanisms such as FcγRI uptake of neoantigens into dendritic cells [54]. However, strong immune responses are typically not achieved with ADC treatment alone, and combination agents, such as checkpoint inhibitors in immunogenic tumors, are often needed.

Our demonstration of the effective combinations of ADCs and ISACs deserves consideration for increasing efficacy in non‐immunogenic tumors. First, we utilize two non‐competing antibodies that can crosslink the CEA receptor and thereby increase internalization and ADC payload efficacy [32]. Our previous work showed similar uptake for fast versus slow internalizing CEA ADCs and demonstrated 20 mg·kg^−1^ was a supersaturating dose. Therefore, the total tumor uptake is not expected to significantly increase, but the intracellular delivery of the ADC could be improved by higher internalization from cross‐linking. Second, to design the combination of ADC and ISAC agents, we tested the effector functions alone and in combination to select the best ISAC antibody. With an enhanced Fc domain, Ab2* (ISAC backbone) had greater Fc effector function (Fig. 2) even in combination with Ab1. It also delivers more immune‐stimulating payload to the immune cells (Fig. 3A). The different FcγR interaction of Ab1 (ADC backbone) and Ab2/Ab2* (ISAC backbone) can be potentially explained by their different binding epitope location. For example, Klein et al. found that differences in epitope binding and higher‐order structural features alter effector functions, and Wang et al. demonstrated that Fab engagement and epitope/antigen context modulate Fc region availability for FcγR [55, 56]. Our results are consistent with the expectation that the Ab2* increases ADCC and ADCP relative to Ab2 through increased binding to low‐affinity Fcγ receptors, but the mutations do not increase immune cell internalization, based on previous research showing the high‐affinity FcγRI drives internalization [48, 57]. When conjugated to a STING agonist at DAR6, the uptake of the ISAC (Ab2*‐STINGa) into immune cells is greater in the presence of cancer cells than without cancer cells, showcasing the increased specificity in ISAC payload delivery (Fig. 3B). This is likely initiated by FcγRI cross‐linking and driving fast immune cell internalization, which has been shown to be important for other STING agonist ISACs [48, 57].

It is also notable that the combination of ADC and ISAC—when using a clinical dose of the ADC (labetuzumab govitecan)—shows the highest efficacy and is the only therapy capable of shrinking the tumors (Fig. 4). Previous work had to dose the mice more frequently than the clinical maximum tolerated dose to achieve tumor regression [32], while the current method inhibits tumor growth using the clinically tolerated ADC dose. Many ADCs show tumor regression in mice at clinically relevant mg·kg^−1^ doses [58], suggesting that the clinically relevant ADC dose in mice can aid in translation. Moreover, the side effects in our study were minor, as the combination therapy caused minor weight loss (~7%), as seen in previous work [23, 24]. While tolerability data in mice have to be interpreted with caution, this minor weight loss was similar to the single ISAC agent alone, indicating the combination is tolerated similarly to the single agents, and there is a lack of additive toxicity in mice. Finally, consistent with the expected mechanism, the STING agonist induced a large influx of immune cells and central tumor necrosis *in vivo* (Fig. 5). The infiltrating cells are likely monocytes and macrophages, as the mice used (*Foxn1* ^*nu*^) are immunodeficient and lack T cells. The central necrosis is often mediated by tumor‐associated macrophages in addition to repolarization of these cells [59, 60]. While the animal model is immunodeficient and cannot generate adaptive immune responses for cures, the overall survival was highest for the combination treatment (Fig. 4). Previous research showed that the ISAC is likely to impact immune signaling much earlier compared to the day 7 harvesting time [48]. However, immune responses from cytotoxic ADC treatment are often slower. Because we were evaluating immune cell infiltration and cancer cell death, we opted for a later time point [49, 50]. Future work will need to analyze the kinetics and immune cell populations using immunocompetent mouse models sensitive to the ADC payload.

The single‐agent efficacy of Ab2*‐STINGa (ISAC) is lower than that for other ISACs such as XMT‐2056 [24]. This agent uses a more potent diABZI payload and higher DAR (DAR8) and can achieve strong tumor regression with single‐agent treatment at 1 mg·kg^−1^ or less. This is consistent with the diABZI being more potent than the CL656 payload in the present study [61]. At the same time, the lower potency of CL656 enabled high doses—namely two doses at 10 mg·kg^−1^—with a similar small reduction in body weight (~7%) [62]. Because the high dose of Ab2 was desirable in this case to match the dose of Ab1‐SN38 (ADC) and induce cross‐linking for efficient tissue penetration and internalization, the lower potency payload was more suitable in this application. Similar to cytotoxic ADCs, the payload potency, DAR, and dosing of the ISAC should be matched to the optimal antibody dose for maximum efficacy [63].

Similar to combination treatment with an ADC and ISAC, dual‐payload ADCs have been tested [64], including cytotoxic and immune‐stimulating payloads on the same antibody [65]. Each approach has advantages and disadvantages. The dual payload approach enables the development and approval of a single agent, providing a more straightforward pathway for development. The development of two separate agents is more complex, but it also has several therapeutic advantages for consideration. Separate antibodies for the cytotoxic and immune‐stimulating payloads enable independent optimization for delivery into cancer and immune cells, respectively. Clinically, a dose reduction due to toxicity from one of the agents can be done without the need to reduce the other therapeutic, tailoring the dose to individual patients. There is also more flexibility in varying each dose to achieve the optimal delivery of both payloads versus a fixed ratio on a single agent. Therefore, the trade‐offs must be weighed when designing these agents.

In this study, the immune infiltration induced by the combination of ADC and ISAC was weaker than that induced by ISAC alone. The result did not reach statistical significance (*P* = 0.068), but it may suggest a meaningful decrease could be occurring. The reason for a potential reduced immune response is unclear, but one hypothesis is that the competition for immune cell FcγRI between Ab1 and Ab2* bound to the cancer cell could attenuate the delivery of Ab2* ISAC into the immune cells. A second hypothesis is that the delivery of the Ab1 cytotoxic payload into immune cells could hamper immune effects. If either effect is occurring, this could be reduced or eliminated by using an effectorless antibody for Ab1, which is another advantage of utilizing two separate antibodies. In the current work, we did not test this for two reasons. First, we wanted to use the clinically tested molecule (labetuzumab govitecan) as the ADC because we know the clinical maximum tolerated dose (MTD) and can use clinically relevant dosing in the mouse model. Changing the Fc region could, in theory, change the clinical toxicity. Second, the choice of Fc region on the ADC is complex, as uptake of cytotoxic drugs into immune cells can sometimes be beneficial by further stimulating immune cells [50]. We utilized non‐overlapping epitopes to increase internalization, but this can also be achieved with a single biparatopic agent [32, 33, 66] to leverage receptor clustering for both strategies. A single biparatopic agent also benefits from lower commercial development costs of one drug versus two. However, the substantial investment in ADCs could yield a significant number of ADC agents suitable for combination treatment. The large number of cytotoxic ADCs currently in clinical trials will determine if and how many wild‐type Fc region ADCs versus Fc‐silent ADCs are available for combination therapy, although all current ADCs in solid tumors are Fc competent. Overall, the advantages of combination therapy must be weighed against the feasibility of designing two agents, which can vary greatly depending on whether a separate ADC or ISAC is approved or under development.

## 5 Conclusion

In conclusion, we developed an ADC and ISAC combination therapy using anti‐CEA antibodies binding non‐overlapping epitopes to increase cancer cell internalization. The Fc/Fc receptor interactions were optimized for increased delivery of the ISAC payload into immune cells. Testing combinations of antibodies with different payloads and Fc effector functions resulted in tumor regression *in vivo* for the combination treatment. These data should help the rational design of ADC and ISAC combination treatments for increased efficacy.

## 7 Conflict of interest

PMT is a member of the scientific advisory boards for Nabla Bio, Aureka Biotechnologies, and Dualitas Therapeutics. GMT has served on the scientific advisory boards of AstraZeneca/Medimmune, Advanced Proteome Therapeutics, Catalent, Merck, Mersana, and Neoleukin.

## 8 Author contributions

PMT and GMT conceived the project. TW and DJK performed the experiments. PMT, TW, and GMT wrote the paper.

## Supporting information


Data S1.

**Fig. S1.** Anti‐CEA antibodies display unique propensities to be internalized into macrophages when co‐cultured with cancer cells.

**Fig. S2.** The Ab2* antibody remains same binding affinity to cancer cells after conjugated to STING agonist.

**Fig. S3.** ISAC displays low *in vitro* toxicity.

**Fig. S4.** The *in vitro* characterization of different CEA expression cell lines.

**Fig. S5.** Tumor volume growth curves for various antibody and control treatments.

**Fig. S6.** Evaluation of mouse weight change following antibody treatments.

**Fig. S7.** Histological imaging of tumors isolated from mice after different treatments.

## Data Availability

All data are reported in this paper or available from the corresponding authors upon request.
