# Supplementary material for: Combining antibody conjugates with cytotoxic and immune‐stimulating payloads maximizes anti‐cancer activity
Source: Mol Oncol. 2026 Jan 6;20(5):1220–36. doi: 10.1002/1878-0261.70198 (PMC13155142; doi:10.1002/1878-0261.70198)
Supplement: Supplementary file 1 — Data S1. [file MOL2-20-1220-s008.docx]

**Figure S1. Anti-CEA antibodies display unique propensities to be internalized into macrophages when co-cultured with cancer cells.** (**A**) Cancer (CAPAN-1) and (**B**) macrophage (J774A.1) cells were mixed at different ratios, seeded in 96-well plates, and fluorescently-labeled IgGs (40 nM each) were added at 37 °C for 4 h. Next, the internalized fluorescent signals were evaluated using flow cytometry (see Methods for more details). (**C**) CAPAN-1 cells were incubated with fluorescently labeled Ab1 and non-fluorescent Ab2* without (no macs) or with a 9:1 ratio of J774A.1 to cancer cells. After 4 hrs, the cells were analyzed by flow cytometry, and the full signal (F, surface and internal) or quenched signal (Q, internal signal only) was measured. The amount of internalized antibody was not statistically significantly different, but there was a small but statistically significant decrease in the total signal, indicating a decrease in the surface antibody (full minus internal). Ab2* is Ab2 with a mutated immune-enhancing Fc. All data are averages of three independent experiments, and the error bars are standard deviations. The statistical significance was evaluated using a paired t-test and *p*-values of >0.05 (ns), <0.05 (*) are indicated on the plots.

**Figure S2. The Ab2* antibody remain same binding affinity to cancer cells after conjugated to STING agonist.** Flow cytometry binding curves of Ab2*-STINGa (ISAC) and Ab2* binding to a CEA-expressing cell line (CAPAN-1). Ab2* is Ab2 with a mutated immune-enhancing Fc. The data are averages of three independent experiments, and the error bars are standard deviations.

**Figure S3. ISAC displays low *in vitro* toxicity.** Cancer (CAPAN-1) cells were seeded into 96-well plates, IgG or ISAC (Ab2*-STINGa) was added at a range of concentrations, and the cells were cultured for four days. Afterward, cell proliferation was measured using a cell titer assay kit. Ab2* is Ab2 with a mutated immune-enhancing Fc. The data are averages of three independent experiments, and the error bars are standard deviations.

**Figure S4. The *in vitro* characterization of different CEA expression cell lines.** (**A**) The CEA expression level on human cancer cell lines (LS174T and CAPAN-1) and mouse cell line (MC38-CEA). The data are averages of three repeats, and the error bars are standard deviations. (**B**) ADC (hMN14-SN38) displays low *in vitro* toxicity to mouse cells (MC38-CEA). Cancer cells were seeded into 96-well plates, ADC was added at a range of concentrations, and the cells were cultured for four days. Afterward, cell proliferation was measured using the cell titer assay kit. The data are averages of four repeats, and the error bars are standard deviations. The same results were observed when the incubation time was two or three days with multiple repeats (data not shown). Antigen negative cells were not tested due to significant payload deconjugation in the media of the CL2A linker, resulting in bystander killing. The statistical significance was evaluated using a paired t-test and *p*-values of <0.01 (**), <0.001 (***), and <0.0001 (****) are indicated on the plots.

**Figure S5. Tumor volume growth curves for various antibody and control treatments.** CAPAN-1 xenografts were used to evaluate the efficacy of antibody treatments. Once the tumor volume reached 250 mm^3^ (day 0), antibody or control treatments were administered via tail vein injection (10 mg/kg for each antibody), and the same dose was administered again on day 7. Ab2* is Ab2 with a mutated immune-enhancing Fc. Each line represents the growth of a single tumor. The blue arrows indicate the date the injection was performed. The tumor volumes were recorded twice a week (n=10).

**Figure S6. Evaluation of mouse weight change following antibody treatments.** Starting from the first IV injection, mouse weights were recorded twice a week. Ab2* is Ab2 with a mutated immune-enhancing Fc. The data are averages, and the error bars are standard errors (n=10).

**Figure S7. Histological imaging of tumors isolated from mice after different treatments.** (**A**-**D**) CAPAN-1 xenografts were applied. Once the tumor volume reached 250 mm^3^, (**A**) PBS, (**B**) ADC (Ab1-SN38), (**C**) ISAC Ab2*-STINGa, and (**D**) ADC (Ab1-SN38) + ISAC Ab2*-STINGa were administered via tail vein injection (10 mg/kg each or 20 mg/kg total for the antibody mixture) on day 0. The tumors were resected and sectioned after 7 days. Tumor slides were stained and imaged using confocal microscopy. CD45 (red, immune cells), HER2 (green, expressed on cancer cells in addition to CEA), and Hoechst (blue) were imaged, and the scale bars are 1 mm. Ab2* is Ab2 with a mutated immune-enhancing Fc. Each image is from a different mouse. *N* stands for necrosis in the tumor.
