# Supplementary figures and images for: Combining antibody conjugates with cytotoxic and immune‐stimulating payloads maximizes anti‐cancer activity
Source: Mol Oncol. 2026 Jan 6;20(5):1220–36. doi: 10.1002/1878-0261.70198 (PMC13155142; doi:10.1002/1878-0261.70198)

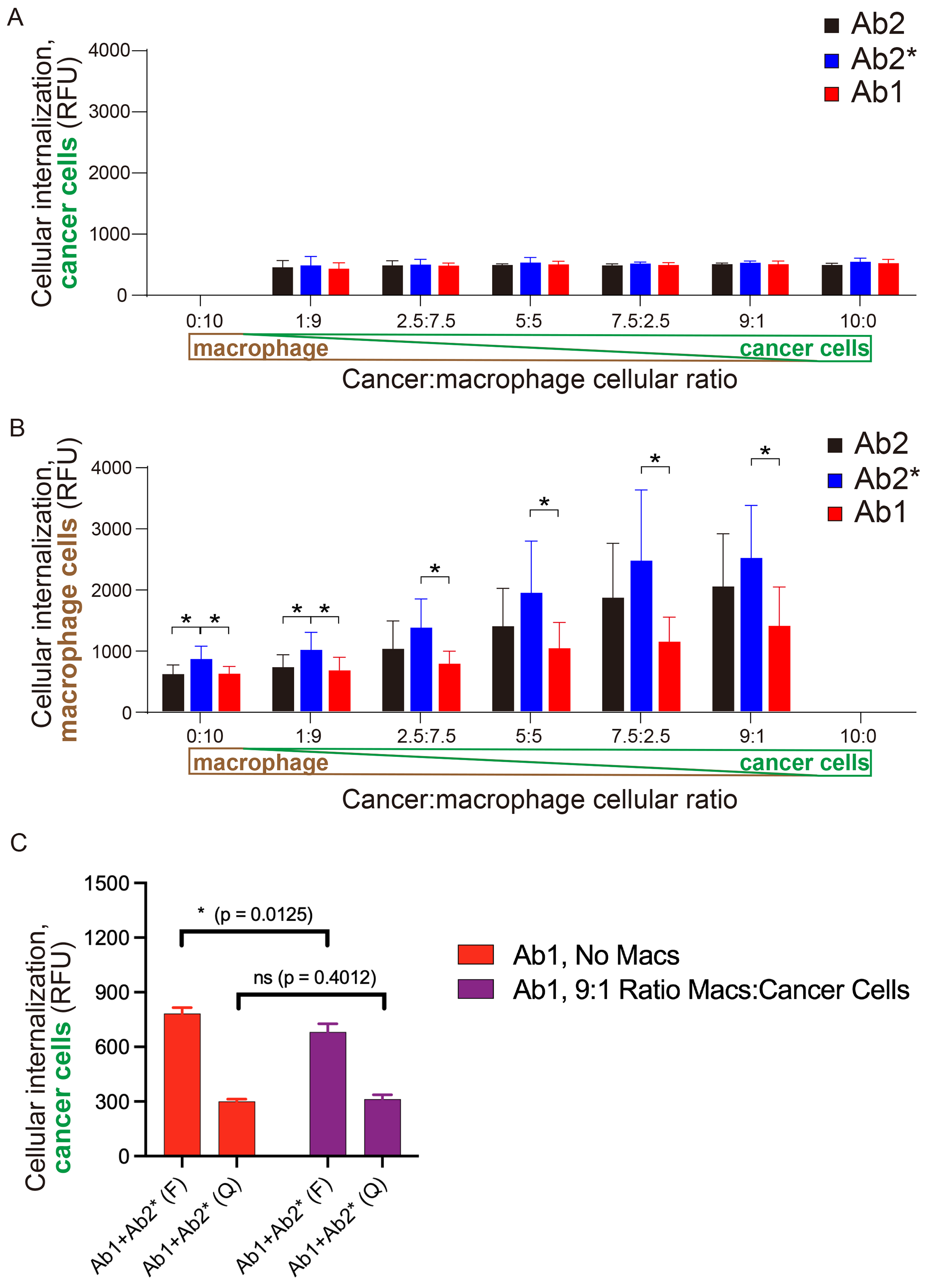

Supplement: Supplementary file 2 — Fig. S1. Anti‐CEA antibodies display unique propensities to be internalized into macrophages when co‐cultured with cancer cells. [file MOL2-20-1220-s003.tif]

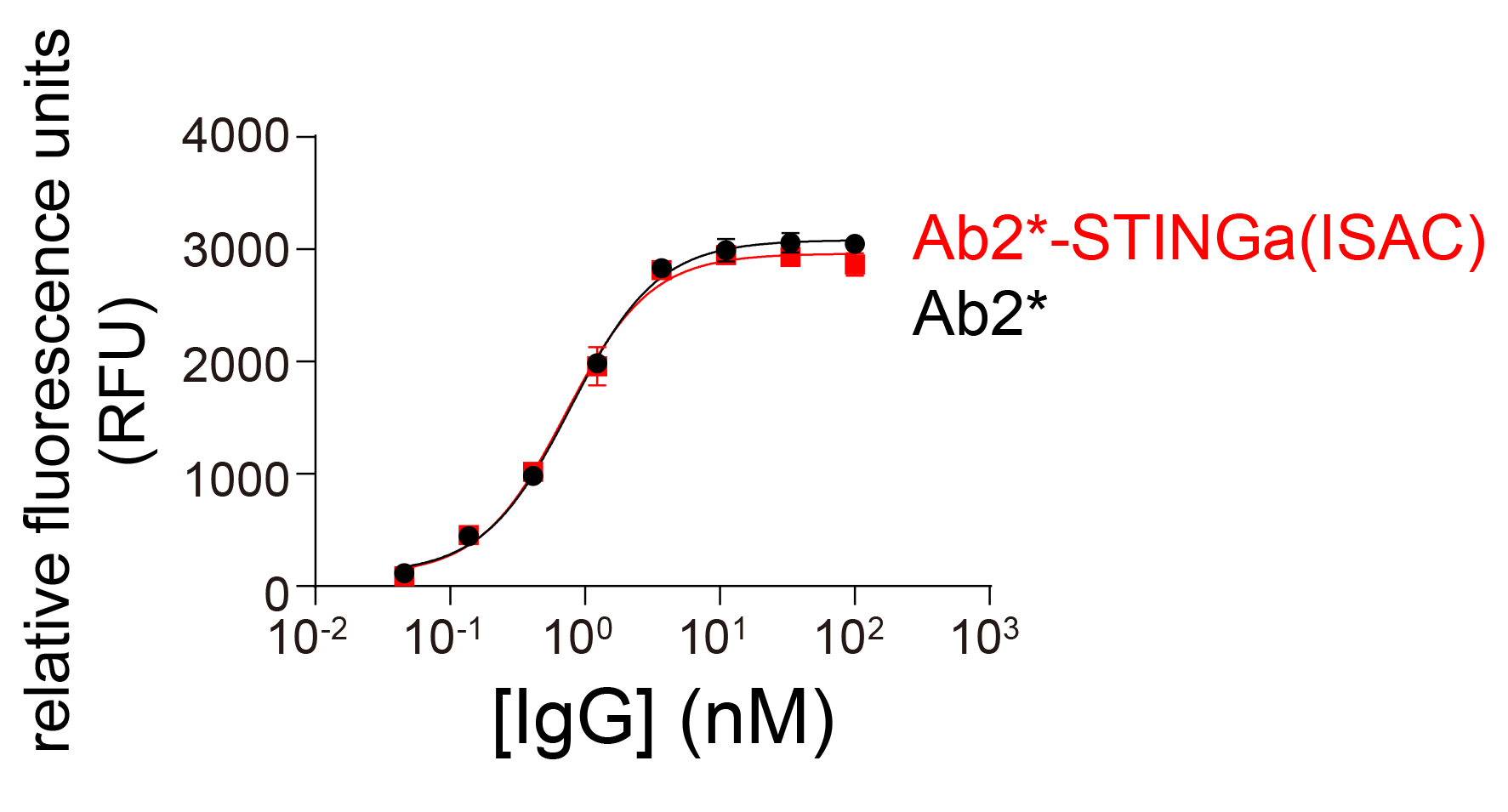

Supplement: Supplementary file 3 — Fig. S2. The Ab2* antibody remains same binding affinity to cancer cells after conjugated to STING agonist. [file MOL2-20-1220-s007.tif]

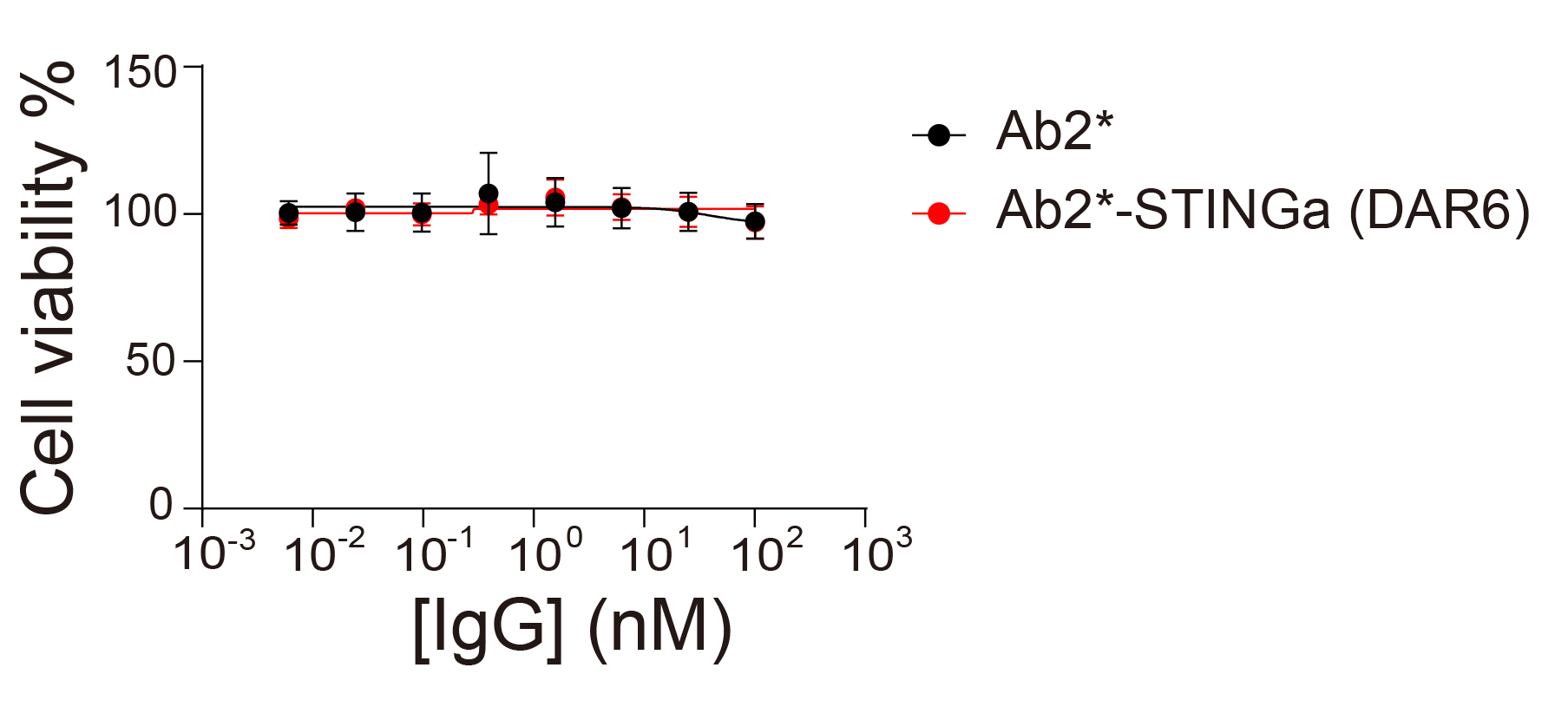

Supplement: Supplementary file 4 — Fig. S3. ISAC displays low in vitro toxicity. [file MOL2-20-1220-s002.tif]

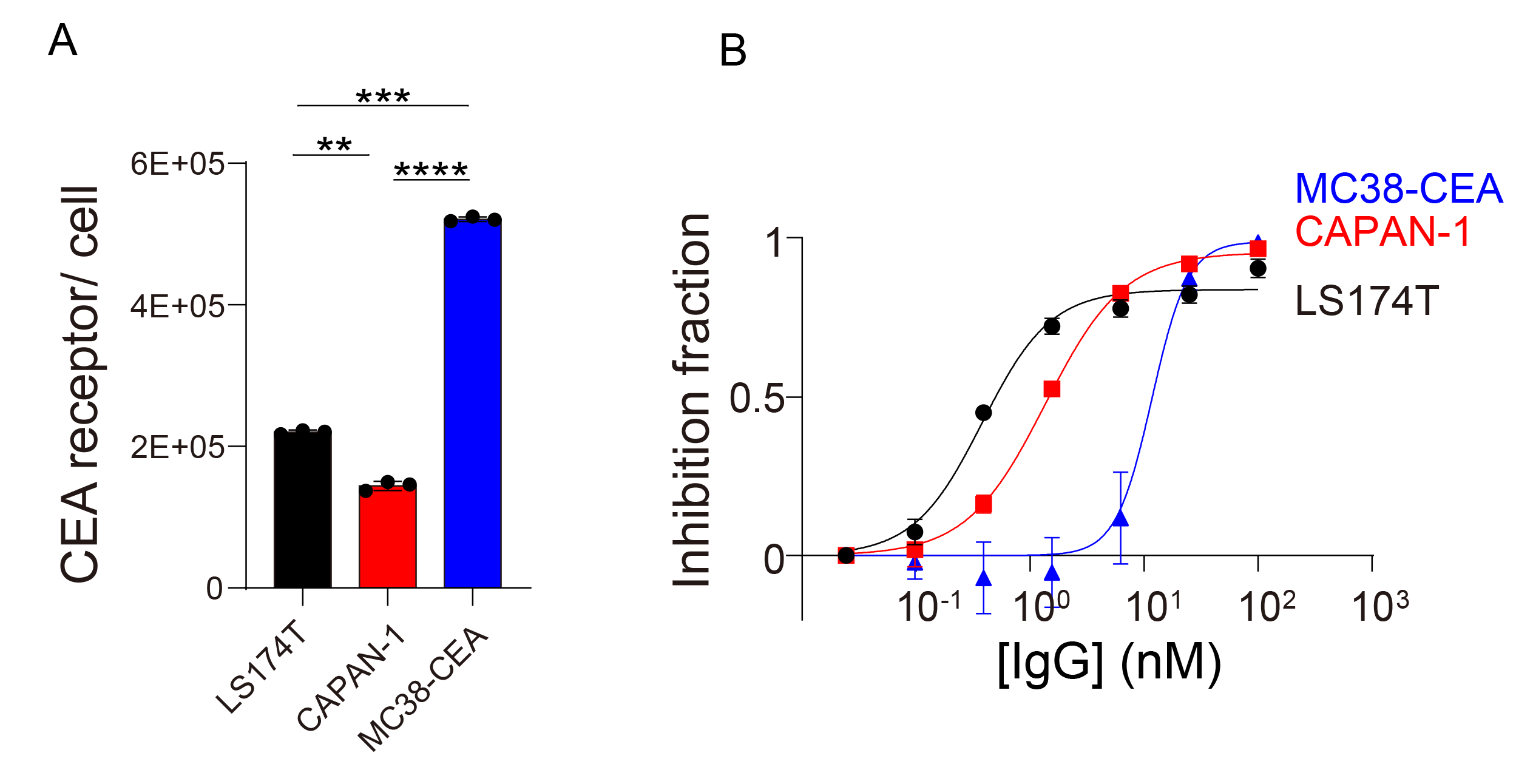

Supplement: Supplementary file 5 — Fig. S4. The in vitro characterization of different CEA expression cell lines. [file MOL2-20-1220-s005.tif]

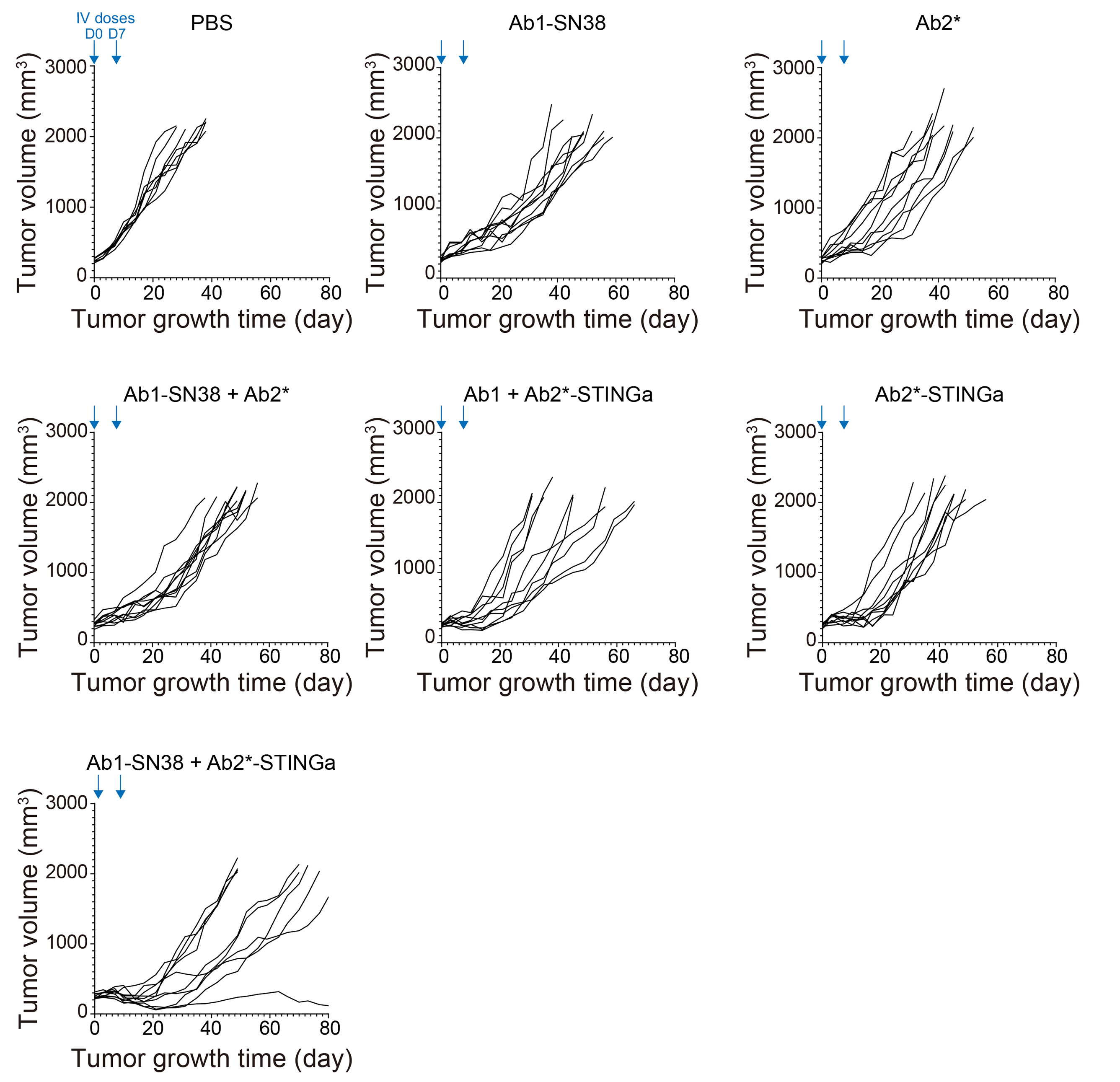

Supplement: Supplementary file 6 — Fig. S5. Tumor volume growth curves for various antibody and control treatments. [file MOL2-20-1220-s001.tif]

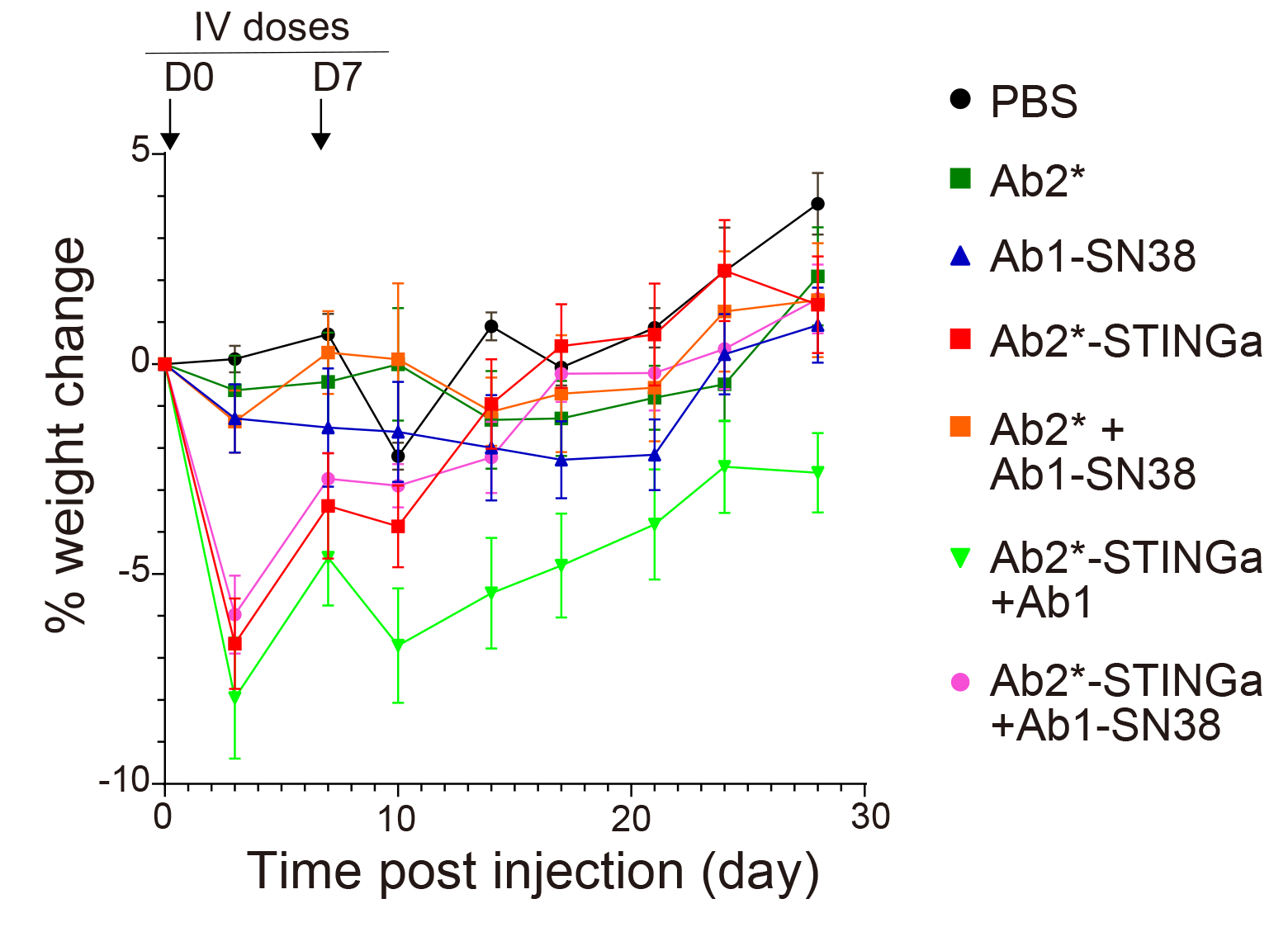

Supplement: Supplementary file 7 — Fig. S6. Evaluation of mouse weight change following antibody treatments. [file MOL2-20-1220-s004.tif]

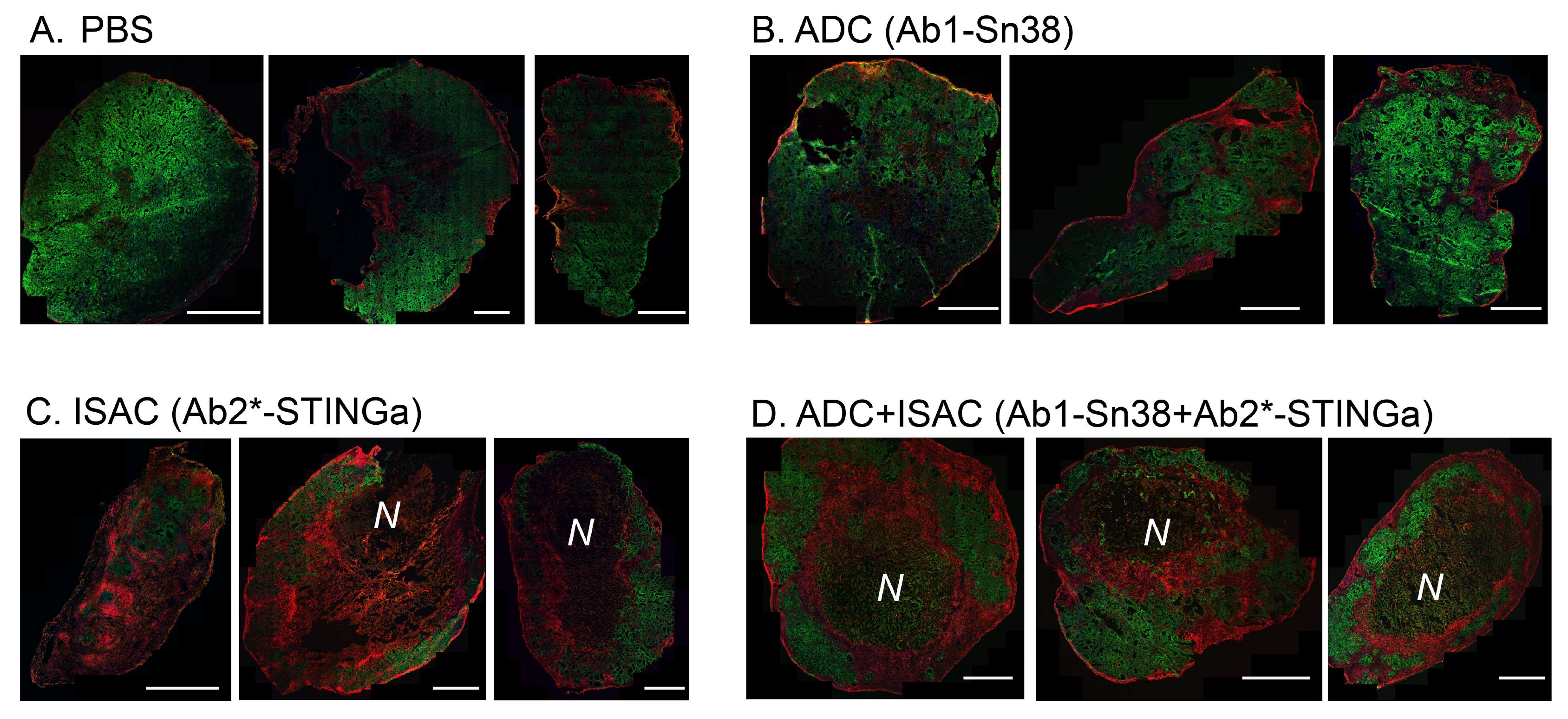

Supplement: Supplementary file 8 — Fig. S7. Histological imaging of tumors isolated from mice after different treatments. [file MOL2-20-1220-s006.tif]
